# A Migraneur with a Usual Headache: A Near-miss of Cerebral Venous Thrombosis

**DOI:** 10.7759/cureus.4393

**Published:** 2019-04-05

**Authors:** Syed Daniyal Asad, Nerea Lopetegui Lia, Bryan W Ferrigno, Hossam Alhabach

**Affiliations:** 1 Neurology, University of Connecticut School of Medicine, Farmington, USA; 2 Internal Medicine, University of Connecticut School of Medicine, Farmington, USA; 3 Neurology, Hartford Hospital, Hartford, USA

**Keywords:** cerebral venous thrombosis, stroke, hypercoagulability, headache

## Abstract

Cerebral venous thrombosis (CVT), while rare, is a challenging diagnosis. It can be easily missed as the presenting symptom can be just a mild headache. However, if missed and left untreated, it can lead to multiple complications, even death. There are certain risk factors that should make one suspect CVT, such as pregnancy, puerperium, use of oral contraceptive pills (OCPs) or known underlying hypercoagulable disorder, to name a few. Imaging is required for diagnosis. Anticoagulation, typically long term, is the standard treatment. We present a case of a 25-year-old male who was initially discharged after an emergency department visit with symptomatic treatment for migraine headaches, and was later found to have extensive cerebral venous sinus thrombosis. It is worth emphasizing the importance of having a broad differential diagnosis and a low threshold to obtain imaging studies when patients present with persistent headaches, even in the absence of any obvious risk factors.

## Introduction

Cerebral venous thrombosis (CVT) is a rare but potentially life-threatening entity. It accounts for only 0.5% of all strokes [1]. It has a varied presentation with headaches, seizures and altered sensorium often mentioned as the classic triad. It can have multiple complications including hemorrhagic infarctions, hydrocephalus, cranial nerve palsies and decreased level of consciousness. Given the lack of unique characteristics, patients who present with intractable headaches, in particular, those with an underlying pro-thrombotic condition, should raise suspicion for CVT.

We report an unusual presentation of CVT which was initially thought to be a typical migraine headache.

## Case presentation

A 25-year-old male with a remote history of migraine headaches without aura in childhood presented to the emergency department (ED) with a three-day history of bitemporal and occipital throbbing headache. The headache was mild in intensity and it was associated with photophobia. It was non radiating. He did not endorse any other symptoms. He was hemodynamically stable. There were no focal neurological findings on exam. His symptoms were attributed to a migraine headache. His headache improved after administration of non-steroidal anti-inflammatory drugs (NSAIDs). He was subsequently discharged home. 

However, he returned to the ED three days later with a persistent headache of similar nature as prior, but this time associated with four hours of intermittent left arm tingling. He did endorse one episode of projectile vomiting a day before this presentation. On further history, he denied dizziness, vertigo, diplopia, visual changes, ear fullness, tinnitus, neck pain, palpitations, weakness or numbness. He had not had a migraine headache since the age of 15 and was not on any medications. Family history was significant for hypertension in his mother and a maternal cousin who passed away at the age of 41 due to a ruptured intracranial aneurysm. There was no family history of hematologic disorders. He was a full-time student and occasionally smoked marijuana.

On examination, the patient’s temperature was 98.4 F, blood pressure was 136/71 mmHg and oxygen saturation at 97% on ambient air. He was a healthy appearing male in no acute distress. Cardiovascular, respiratory, abdominal, and skin examination was unremarkable. Neurological exam did not reveal any cognitive, language, cranial nerve, motor or sensory deficits. Gait was normal. The patient’s complete blood count with differential and basic metabolic panel were unremarkable.

However, the patient kept complaining of severe photophobia, headache, and intermittent left arm tingling. He did not experience relief with intravenous pain medications. Given his second visit to the ED and symptoms of intermittent left arm tingling, it was decided to order a computed tomography (CT) of the head without contrast.

**Figure 1:**
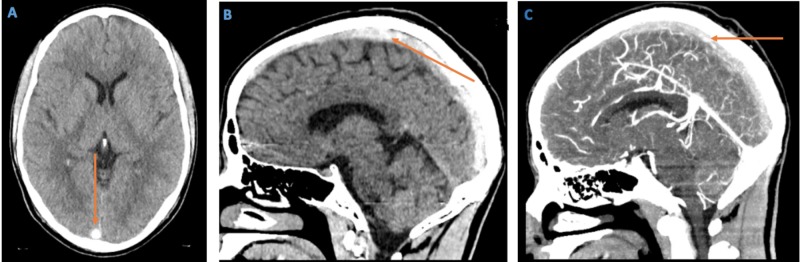
Computed tomography (CT) of the head without contrast (axial and sagittal views) and CT angiogram of the head A) CT head without contrast (axial view) showing hyperdensity at the torcula; B) CT head without contrast (sagittal view) showing hyperdensity at the superior sagittal sinus; C) CT angiogram (brain window) demonstrates extensive hyperdensity in the superior sagittal sinus.

CT head without contrast, as seen in Figures 1A-1B, unexpectedly revealed a well-circumscribed hyperdensity at the inion corresponding to the torcula as well as a prominent hyperdense right transverse sinus. Therefore, CT angiogram of head and neck vessels was performed which showed extensive CVT of the superior sagittal sinus, right transverse sinus with extension into the sigmoid sinus, and jugular bulb (Figure 1C).

**Figure 2:**
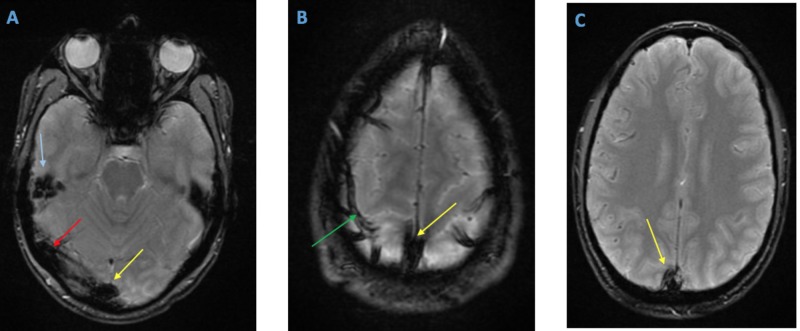
Magnetic resonance imaging (MRI) of the brain - axial gradient recall echo (GRE) sequence A, B, and C) MRI of the brain without contrast (GRE sequence, axial view) shows susceptibility changes demonstrating thrombosis of the superior sagittal sinus (yellow arrows), sigmoid sinus (blue arrow), right transverse sinus (red arrow), and cortical veins (green arrow).

There was also involvement of the cortical veins with evidence of trace subarachnoid hemorrhage in the left frontal sulcus. Magnetic resonance imaging (MRI) of the brain without intravenous contrast confirmed these findings (Figure 2).

Hypercoagulability panel was sent which included partial thromboplastin time, prothrombin time, antithrombin III activity, protein C activity and antigen, protein S activity and antigen, Anti-nuclear antibody screen, serum beta-2 glycoprotein antibodies (IgA, IgM, IgG), serum cardiolipin antibodies (IgA, IgM, IgG), lupus anticoagulant with reflex to dilute Russell’s viper venom time (dRVVT), serum homocysteine level, lipoprotein (a) and sickle cell screen. Genetic analysis for mutations or polymorphisms in methyl tetrahydrofolate reductase (MTHFR) gene, factor II prothrombin gene, and Factor V Leiden gene were also tested.

Subsequently, the patient was started on a full dose of heparin infusion with goal anti-Xa level between 0.3-0.7 IU/mL. The patient had one generalized tonic-clonic seizure the day after admission for which he was started on levetiracetam. His neurological exam and repeat CT head without contrast remained stable.

The hypercoagulable panel was only notable for slightly elevated homocysteine level at 13.8 umol/L (normal <11.4 umol/L) and one copy of the c.665C>T GenBank#NM_005957.4 (previously known as C677T polymorphism in the MTHFR gene). Serum folic acid was normal. Further work up including CT of the chest, abdomen and pelvis, transthoracic echocardiogram, venous duplex ultrasound of the lower extremities were all unremarkable.

The patient was started on long-term anticoagulation with warfarin with goal international normalized ratio (INR) between 2.0 and 3.0 and discharged home with plans for outpatient follow up.

## Discussion

CVT can be a life-threatening condition with a myriad of complications. Risk factors for the development of CVT can be divided into transient and chronic. Transient risk factors include pregnancy, puerperium, use of oral contraceptive pills (OCPs), dehydration, prolonged travel; while chronic risk factors include hereditary or acquired thrombophilias [2] An international retrospective study spanning 21 countries by Ferro and colleagues titled “International Study on Cerebral Venous Thrombosis” (ISCVT) provided a lot of insight into the prognosis and presentation of CVT [3]. Isolated headache is rare (~ 15%) and is often associated with other signs of intracranial hypertension like diplopia or decreased level of consciousness [3-4]. Headache can be unilateral, throbbing or sometimes thunderclap but photophobia is rarely reported [5].

International Classification of Headache Disorders (ICHD-3) described these headaches as diffuse, progressive and severe, and associated with other signs of intracranial hypertension. However, sometimes CVT can present as sudden onset unilateral headaches, mimicking migraine headache (with or without aura), cluster headache, hemicrania continua, primary thunderclap headache, a non-traumatic subarachnoid haemorrhage or a headache attributed to low cerebrospinal fluid pressure [6].

According to the ISCVT study [2], 85% of those diagnosed with CVT had at least one risk factor which includes prothrombotic states, pregnancy, use of OCPs. Our patient only had mildly elevated homocysteine levels at 13.8 umol/L (normal <11.4 umol/L) and one copy of the c.665C>T GenBank#NM_005957.4 (previously known as C677T polymorphism in the MTHFR gene). Heterozygosity for the c.665C>T mutation is not associated with increased homocysteine plasma levels and is not associated with increased risk of thrombosis. At this time, homozygosity for polymorphism c.665 C>T (C677T) is the only MTHFR genotype that has been shown to be clinically significant. These genotypes are associated with a mild reduction in MTHFR enzyme activity, and an elevated plasma homocysteine level, particularly when plasma folate is low [5,7].

Neuroimaging is needed to reach the diagnosis: MRI with T2*-weighted images in addition to magnetic resonance angiography (MRA), or CT scan plus CT angiography, as well as intra-arterial angiography in selected cases [6].

Multiple aspects of this case are interesting. Firstly, the continued isolated dull headache with photophobia understandably led the providers to initially think of it as a migraine headache. Photophobia with headache is not usually seen in CVT as discussed above. What stood out in the history was the fact that the patient had not had a persistent headache for many years despite having been diagnosed with migraine headaches in his adolescence. This raised the suspicion of another pathology at play. Secondly, the patient had an excellent neurological outcome despite such extensive thrombosis without common complications such as hydrocephalus and hemorrhage. While venous infarctions and hemorrhage is present in up to 35%-39% of cases with such extensive involvement of the dural venous sinuses [2], our patient only had a small focus convexity subarachnoid hemorrhage in one sulcus. Moreover, our patient only had slightly elevated homocysteine levels with normal plasma folate. While hyperhomocystenemia does increase the risk of thrombosis, it is surprising to see this as the only abnormality potentially predisposing our patient to CVT especially given normal serum folate levels.

Treatment of CVT usually requires long term anticoagulation [7]. However, new therapies are being studied and used including thrombolysis and mechanical thrombectomy [8-9], which is beyond the scope of this case report.

## Conclusions

While headaches in young people without focal deficits are usually benign and can be treated symptomatically, healthcare providers, especially in the ED, should keep CVT on the list of differential diagnoses. If the headache is persistent and not responsive to standard symptomatic therapy, imaging should be used to help guide diagnosis.
